# Effect of inorganic nitrogen concentration in co-culture and regeneration media on *Agrobacterium tumefaciens* growth and on the regenerative capacity of transformed *Pinus radiata *embryonal mass

**DOI:** 10.1186/1753-6561-5-S7-P141

**Published:** 2011-09-13

**Authors:** Regis Le-Feuvre, Claudia Triviño, Ana María Sabja, Krystyna Klimaszewska

**Affiliations:** 1 Genomica Forestal S.A., CBT Universidad de Concepción, Concepción, Chile; 2 Genfor S.A., Fundación Chile, sucursal Valdivia, Valdivia, Chile; 3 Natural Resources Canada, Canadian Forest Service, Laurentian Forestry Centre, 1055 du P.E.P.S., Quebec, QC G1V 4C7, Canada

## 

The Genetic Engineering program at Genomica Forestal SA, Chile (GFSA) has a goal of generating stably transformed radiata pine for *in planta* evaluation of candidate genes. Regeneration of transgenic plants depends mainly on two factors: regeneration ability of transformed cells and stable transgene integration and expression.

In several conifer species, including radiata pine, transgenics have been regenerated through cocultivation of *Agrobacterium tumefaciens* with embryogenic cells [1-3]. However, in our first experiments using MSG [4] culture medium we found that radiata pine embryonal masses did not recover easily after co cultivation and that there was an excessive overgrowth of bacterial cells in spite of using bacteriostatics in the medium. This impediment prompted our study on testing other culture medium formulations, routinely used in conifer somatic embryogenesis, on the growth of *A. tumefaciens* GV3101. The tested media were: MSG, DCR [5], and modified Litvay [6] MLV. Of the three media MSG supported significantly higher bacterial growth than the other two media. One of the major differences in the composition of these media is inorganic nitrogen concentration (NH_4_NO_3_ and KNO_3_). Compared with MLV and DCR, MSG has the lowest concentration of inorganic nitrogen (100 mgl^-1^ compared with 340 in DCR and 950 mgl^-l^ in MLV) provided in the sole form of KNO_3_. Based on our results and the work of others, we concluded that low nitrate concentration in MSG medium promoted *A. tumefaciens* growth and this had a deleterious influence on the viability of radiata pine cells during co cultivation, and also rendered eradication of bacterial cells difficult. Comparison of growth of radiata pine embryonal mass on the three media did not show statistically significant differences. A strategy for producing transgenic radiata pine for *in planta* transgene expression and stability study will be presented.
